# Low‐dose oral isotretinoin in the treatment of recalcitrant facial flat warts: A clinical case and review of literature

**DOI:** 10.1002/hsr2.1633

**Published:** 2023-10-25

**Authors:** María Guadalupe Olguín‐García, María Luisa Peralta‐Pedrero, Fermín Jurado‐Santa Cruz, Elisa Vega‐Memije, Martha Alejandra Morales‐Sánchez

**Affiliations:** ^1^ Universidad Nacional Autónoma de México “Programa de Maestría y Doctorado en Ciencias Médicas, Odontológicas y de la Salud” Mexico City Mexico; ^2^ Education and Research Department Centro Dermatológico Dr. Ladislao de la Pascua (CDP) Secretaría de Salud de la Ciudad de México Mexico City Mexico; ^3^ Internal Medicine Department Hospital General Dr. Darío Fernández Fierro, ISSSTE Mexico City Mexico; ^4^ Dermatology Department Hospital General Dr. Manuel Gea González Secretaria de Salud Mexico City Mexico

**Keywords:** low‐dose isotretinoin, recalcitrant flat warts, treatment

## Abstract

**Background and Aims:**

The treatment of recalcitrant facial flat warts has always been challenging for dermatologists. The pain related to the application of the different treatments, side effects and costs are determining factors in the choice of therapy. To date, it is known that oral isotretinoin administered at a dose of 0.5 mg/kg/day is effective and safe; However, the different adverse effects reported have a dose‐dependent behavior and they could limit their use. Our aim is to assess the effect of low‐doses of oral isotretinoin to reducing side effects in the complete removal of recalcitrant facial flat warts and the current evidence in this regard.

**Methods:**

An extensive literature review was conducted to identify articles relating to low doses of oral isotretinoin for recalcitrant flat warts treatment, regardless of design up to May 2023.

**Results:**

The literature search yielded eight articles of 324 reviewed meeting criteria. Isotretinoin was administered in doses of 0.1–0.5 mg/kg/day. Complete elimination of the lesions occurred in 65.13% of the patients and a partial response in 19.26%. Four relapses were documented at the 4‐month follow‐up. The most frequent adverse effect was cheilitis.

**Conclusion:**

We might consider low doses of oral isotretinoin for the treatment of recalcitrant facial flat warts in which side effects need to be reduced. However, current published works have several limitations, including small sample sizes, lack of control group and follow‐up periods. Larger, randomized, controlled studies are needed to verify the efficacy and safety of different doses of isotretinoin.

## 1 BACKGROUND AND AIMS

Facial flat warts are an infectious and common cosmetic problem in patients seeking dermatologic care. Flat warts are growths on the skin, caused by subtypes 3 and 10 of the human papillomavirus (HPV). They usually appear in children and adolescents, but can occasionally be found in adult women and human immunodeficiency virus (HIV) patients, especially on the face and back of the hands. Clinically, they are observed as smooth papules of 2 to 4 mm in diameter, pigmented, frequently multiple, sometimes presenting Koebner's phenomenon and can spontaneously regress up to two thirds of cases within 2 years.^1^

Although there are several therapies available: destructive (cantharidin, salicylic acid); virucidal (cidofovir, interferon‐α); antimitotic (bleomycin, podophyllotoxin, 5‐fluorouracil); immunotherapy (Candida antigen, contact allergen immunotherapy, imiquimod); several (trichloroacetic acid, polyphenon E.)^2^ is notoriously difficult to treat and up to 30% of cases can become recalcitrant.^3^

The cost, the pain related to the application of different treatments and side effects like scars or disagreeing lesions are determining factors in the choice of therapeutics, which is why treating recalcitrant facial flat warts has always been a challenge for dermatologists.^4^ To date, it is known that oral isotretinoin administered at a dose of 0.5 mg/kg/day for 12 weeks is effective and safe for the treatment of recalcitrant facial flat warts.^5^ Even with this dose, which is already considered low, different adverse effects reported like cheilitis, tiredness, nose bleeds, muscle aches and eyes problems are observed; because all adverse effects have a dose‐dependent behavior.^6^ It is recommended to reduce the dose of isotretinoin in addition to changes in lifestyle and diet in case of increased levels of alanine aminotransferase (ALT), aspartate aminotransferase (AST), triglycerides, cholesterol and creatine phosphokinase (CPK).^7^, ^8^ Although the doses used for the treatment of recalcitrant facial flat warts are considered low,^9^ two cases are reported where the doses of isotretinoin were further decreased (0.3−0.4 mg/kg/day); In both patients the lesions disappeared and there were no side effects.^10^

To find out if at low doses of isotretinoin, it is possible to reduce the adverse effects without affecting the efficacy, we present a case and perform an exhaustive review of the literature to assess the current evidence on the use of low doses of isotretinoin in the treatment of recalcitrant flat facial warts.

### 1.1 Clinical case

**Figure 1:**
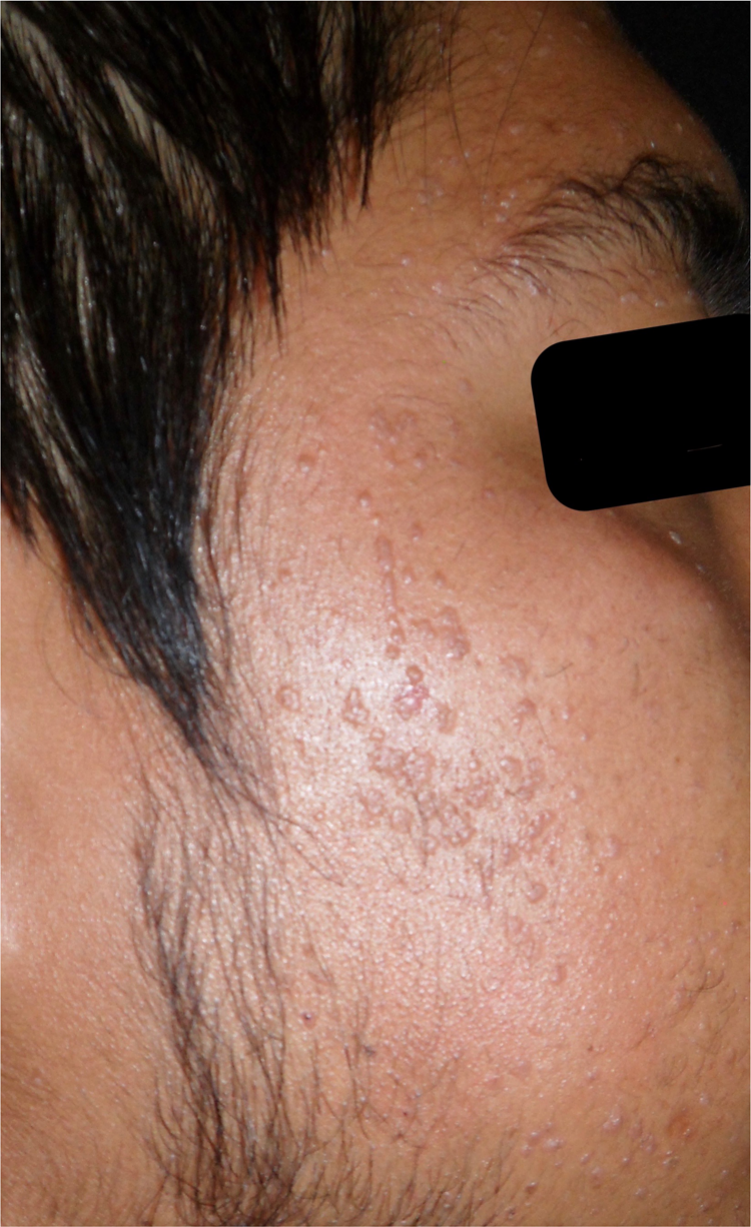
Multiple recalcitrant facial flat warts before treatment with oral isotretinoin.

A 27‐year‐old man with flat warts on her face, predominantly in the frontal region, cheeks and chin of 6 years of evolution, came to our attention. (Figure 1).

**Figure 2:**
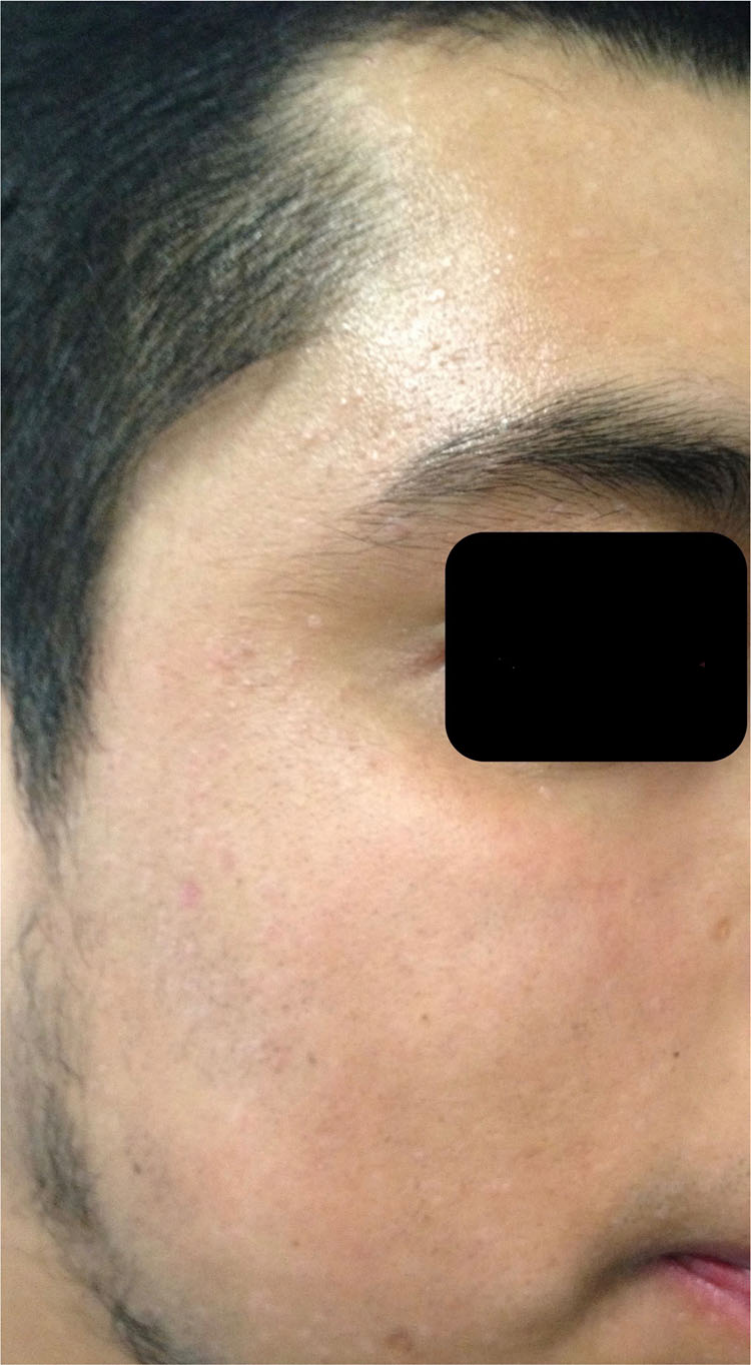
After treatment with isotretinoin at 30 mg/day for 4 weeks and 15 mg/day for 8 weeks.

He initially applied 0.1% tretinoin for 7 months and then 5‐fluorouracil and 5% imiquimod, she went to cryotherapy on three occasions; without response to the treatments used and with the development of spots as well as two residual scars. Oral isotretinoin was prescribed at 0.5 mg/kg/day (30 mg per day), with which the lesions began to disappear. After a month of treatment, the patient referred asymptomatic and satisfied with the evolution; however, the transaminases and creatine phosphokinase (CPK) increased four4 levels above baseline; as well as cholesterol at 300 mg/dl and triglycerides at 310 mg/dl. We agreed to cut the isotretinoin dose in half to 15 mg/day. He was evaluated by Internal Medicine. The patient finished 3 months of treatment, he tolerated it well and a decrease in cholesterol, triglycerides, transaminases and CPK levels observed. To date, no injuries or complications have been observed. (Figure 2).

## 2 METHODS

An extensive literature review was conducted using PubMed, Ovid, LILACS, Scopus, and Cochrane's CENTRAL database to identify articles relating to patients with flat warts and/or recalcitrant flat warts treated with low doses of oral isotretinoin. The search was conducted for the following terms: “isotretinoin” AND “flat warts” OR “plane verruca” with the corresponding subject headings, from the start date of the databases until May 2023.

The search yielded 324 total results. Two independent reviewers (OG and PP) manually screened the articles independently for eligibility. Due to the few controlled clinical trials, any experimental or observational study of case series, even case reports as well as review articles were included. To increase sensitivity, the search terms were deliberately kept broad, including all clinical studies and all available results. No language restrictions were established. Duplicated studies, review articles, related to other types of warts, verruciform epidermodysplasia and immunocompromised were excluded. After the selection process, 20 publications were eligible for full‐text review. Of these, only eight met the inclusion criteria, three of which were clinical trials, three case series with more than 10 patients included, and two case reports. Twelve articles were excluded, a letter to the editor, three classical review articles, four case reports of transplanted patients, and four were trials for the treatment of genital and common warts.

## 3 RESULTS

### 3.1 Demographics and clinical features

A total of 150 patients with flat warts were included in the selected studies (Table 1). The patients were predominantly female except in two studies.^11^, ^12^ Patients ranged from age 4 to 55; three of these studies included children^9^, ^11^, ^13^ and the duration of warts ranged from 1 to 84 months. Face was the most commonly involved site; other types of warts were considered in one study.^11^

**Table 1:** Characteristics of included studies.

Authors, country, year, design	Sample size, sex, age (years)	Type warts, Evolution, Number of treatments	Intervention and duration	Response	Adverse effects	Losses	Follow up	Relapse
(5) Olguín‐García	*n* = 31	Recalcitrant facial	G1 = 16	G1= Complete	Cheilitis	None	uninformed	uninformed
Mexico 2014	m = 10	flat	OI 0.5 mg/kg/day		Dry skin			
Clinical trial	f = 21	>4 years	G2 = 15					
	Age = 18–40	>4	Placebo	100%	Dry eyes			
		treatments	12 weeks	G2 = None^b^	Photosensitivity			
					Pruritus			
					Headache			
					Muscle pain			
(12) Kaur	*n* = 40	Multiple plane warts	G1 = 20	G1 = 16	G1	G1 = 4	4 months	G1=
India	m = 21	5–17 months	OI 0.5 mg/kg/day	Complete 69%	Mild cheilitis	2 = severe cheilitis		36%
2017	f = 19		G2 = 20	Partial 31%	Severe cheilitis	1 = polymenorrhea		G2=
Clinical trial	Age = 21–55		IT 0.05% once per night	G2 = 13	Xerosis	G2 = 5		40%
			3 months or until complete clearance	Complete 38%	Menstrual irregularity	Severe erythema and scaling		
				Partial 46%	G2			
				None 15%	Mild erythema and buming sensation			
					Severe erythema and scaling			
(13) Nofal	n = 108	Plane	G1 = 36	G1: Complete 44.4%	OI	None	6 months	None
Egypt 2020	m = 48	1–36 months	OI 0.3 mg/kg/day	Partial 44.4%	Cheilitis and dry skin			
Clinical Trial	f = 60		3 months	None 11.2%	Ca			
	Age = 4–44		G2 = 36	G2: Complete 55.6%	Tolerable pain			
			Ca 0.1 ml of 1/1000 solution	Partial 11.1%	Edema/induration			
			at 2‐week intervals until complete clearance or for maximum of 5 sessions	None 33.3%	Erythema			
			G3 = 36	G3: Complete 38.8%	Flu‐like symptoms			
			OI + Ca	Partial 55.6%				
			same doses	None 5.6%				
			same time					
(14) Olguín‐García	n = 12	Recalcitrant Facial Flat	OI 0.5 mg/kg/day	Complete	Cheilitis	One	6 months	None
Mexico 2010	m = 3	3–7 years	12 weeks = 3 patients	92%	Dry skin			
Cases	f = 9	>4 treatments	10 weeks = 3 patients		Dry eyes			
serie	Age = 18–25		8 weeks = 4 patients					
			6 weeks = 1 patients					
(9) Al‐Hamamy	n = 31	Resistant plane	OI 0.5 mg/kg/day	Complete 73.07%	Dry lips	Five	4 months	None
Iraq	m = 11	8–48 months	2 months	None	Dry skin			
2012	f = 21			26.92%	Epistaxis			
Cases	Age = 5–35				Dry nose			
serie					Headache			
(11) Dave	n = 14	Refractory Plane, common, venereal	OI	Complete 100%	Cheilitis	None	3 years	None
India								
2019	m = 10	1‐36 months	0.1–0.2 mg/kg/day					
Cases	f = 4		3 months					
serie	Age = 7–52							
(10) Miljkovic	n = 2	Multiple facial plane	OI	Total clearance 100%	None	‐‐‐‐‐‐‐‐	6–12 months	None
Slovenia 2012	m = 1	1–2 years	m = 0.3 mg/kg/day					
Case report	f = 1	5 treatments	f = 0.4					
	Age = 40/21		mg/kg/day					
			1 month					
(15) Biatecka	n = 1	Plane	OI	Complete	Dry/exfoliation lips	‐‐‐‐‐‐‐‐‐	uninformed	None
Poland	female	Several months	0.5 mg/kg/day		Dry eyes			
2017	31 years	7 treatments	3 months					
Case report								

^a^ Placebo group received 0.5 mg/kg/day of isotretinoin for 12 weeks after the end of the trial, all them had complete response.

Abbreviations: Ca, antigen Candida; f, female; G, group; m, male; n, sample size; OI, Oral isotretinoin; TI, Topical isotretinoin.

### 3.2 Studies features

The studies analyzed were prospective: three clinical trials, three case series, and two case reports Comparative studies included placebo, topical isotretinoin, and Candida antigen.^5^, ^12^, ^13^ Only two carried out a histological study for diagnostic confirmation.^5^, ^14^

### 3.3 Isotretinoin doses

The doses of isotretinoin used were 0.1–0.5 mg; 75 patients received 0.5 mg, 73 patients 0.3 mg, one 0.1 and another 0.4 mg/kg/day. The time of treatment ranged from 1 to 3 months; 122 received the treatment for 3 months, 26 2 months and two only carried it out for 1 month. Except for two studies,^5^, ^14^ all referred to have used low doses of oral isotretinoin.

### 3.4 Clinical efficacy

In the comparative studies, the treatment was administered for 12 weeks, in two of them (against placebo and isotretinoin gel respectively) the dose used was 0.5 mg/kg/day^5^, ^12^ and in the one compared against candida antigen it was 0.3 mg/kg/day.^13^ Two studies assessed the efficacy of 0.5 mg/kg/day, in the first one the complete response (CR) was observed in 31 (100%) patients; in the second trial the complete response was assessed in 11 (69%) patients. In this study, five patients (31%) had partial response (PR). (Table 1).

Two of the three descriptive studies were conducted for 3 months of treatment,^11^, ^14^ one at a dose of 0.5 mg/kg/day and the other at a dose of 0.1 mg/kg/day; with a CR in the 12 (100%) patients included. In the third study, patients received 0.5 mg/kg/day, but only for 8 weeks; CR was observed in 19 (73.07%) patients and PR in 7 (26.92%) patients.^9^

In these trials, 95 patients were treated with isotretinoin at 0.5 mg/kg/day, 76 of them (80%) had CR, 5 (5.3%) PR, 8 (8.4%) had no response to treatment and 5 (5.3%) were lost to follow‐up. In the case reports, 0.5 mg/kg/day^15^ weight was used in a patient for 3 months, who presented CR. (Table 1).

We found three studies who assessed the efficacy of isotretinoin at 0.3 mg/kg/day in the treatment of flat warts. In the first study, 16 patients (44.4%) had CR, 16 (44.4%) PR and 4 had no response to therapy.^13^ In the second study of two patients, all of them (100%) had CR.^10^ Of these patients, one received 0.4 mg/kg/day of isotretinoin. In another study, one patient with flat warts had CR with 0.1–0.2 mg/kg/day of isotretinoin.^11^ In summary, 37 patients were treated with isotretinoin at 0.3 mg/kg/day, one with 0.4 mg/kg/day and other with 0.1–0.2 mg/kg/day. Nineteen (48.7%) patients had CR, 16 (41%) PR and 4 (10.2%) had no response to treatment with these doses of isotretinoin. (Table 1).

### 3.5 Adverse effects, follow‐up and quality of life

The side effect appeared in 100% of the cases is cheilitis, followed by dry skin 69.42%. In all studies, cheilitis is described as completely tolerable with emollients, except in two patients who received 0.5 mg/kg/weight of isotretinoin, who had to discontinue the drug because the cheilitis was severe.^12^

In the studies where oral isotretinoin was used at a dose of 0.5 mg/kg/day, different side effects were described: dry eyes 18.47%; dry nose 13.37%; photosensitivity 10.19%; pruritus, facial erythema and edema 7.64%; weight loss 6.36%; headache 5.09%; rash 1.91% and muscle pain 1.91%; impaired vision, diffuse alopecia and epistaxis 1.27%.^5^, ^9^, ^12^, ^14^, ^15^

In those patients taking doses between 0.1 and 0.4 mg/kg/day of isotretinoin, except for tolerable cheilitis, no other side effects were described. All patients tolerated the drug and no losses in the follow‐up were reported.^10^, ^11^, ^13^

Regarding the follow‐up time at the end of the intervention, it was 6–36 months. Only one study reported recurrence in four patients who had had complete remission of the lesions at 4 months of follow‐up.^12^ In the others, no relapses were reported. The quality of life of the patients before or at the end of the intervention was not assessed in any of the reviewed studies.

## 4 CONCLUSIONS

We might consider low doses of oral isotretinoin for the treatment of recalcitrant facial flat warts in which side effects need to be reduced. However, current published works have several limitations, including small sample sizes, lack of control group and follow‐up periods. Larger, randomized, controlled studies are needed to verify the efficacy and safety of different doses of isotretinoin. We recommend in future clinical trials to include patients with recalcitrant flat warts and assess the clinical response by age.

## AUTHOR CONTRIBUTIONS

**María Guadalupe Olguín‐García**: Conceptualization; Data curation; Formal analysis; Investigation; Methodology; Project administration; Resources; Validation; Writing—original draft; Writing—review & editing. **María Luisa Peralta‐Pedrero**: Formal analysis; Investigation; Methodology; Project administration; Supervision; Validation; Writing—review & editing. **Fermín Jurado‐Santa Cruz**: Validation; Visualization. **Elisa Vega‐Memije**: Resources; Supervision; Validation; Visualization. **Martha Alejandra Morales‐Sánchez**: Conceptualization; Formal analysis; Methodology; Supervision; Validation; Visualization.

## CONFLICT OF INTEREST STATEMENT

The authors declare no conflicts of interest.

## ETHICS STATEMENT

I, on behalf of all co‐authors, state that the work being submitted has been done in accordance to Wiley's Best Practice Guidelines on Publishing Ethics, in an ethical and responsible way, with no research misconduct, which includes, but is not limited to data fabrication and falsification, plagiarism, image manipulation, unethical research, biased reporting, authorship abuse, redundant or duplicate publication, and undeclared conflicts of interest and all authors have read and approved the final version of the manuscript. Informed consent has been obtained from the patient. No personal information that could identify the patient is included.

## TRANSPARENCY STATEMENT

The lead author María Luisa Peralta‐Pedrero affirms that this manuscript is an honest, accurate, and transparent account of the study being reported; that no important aspects of the study have been omitted; and that any discrepancies from the study as planned (and, if relevant, registered) have been explained.

## Data Availability

The authors confirm that the data supporting the findings of this study are entirely available within the article.
